# Theoretical Studies on the Electronic Structure Parameters and Reactive Activity of Neu5Gc and Neu5Ac under Food Processing Solvent Environment

**DOI:** 10.3390/molecules24020313

**Published:** 2019-01-16

**Authors:** Rui Chang, Bowen Yang, Qiu-Jin Zhu

**Affiliations:** 1 School of Liquor and Food Engineering, Guizhou University, Guiyang 550025, China; cr2011lz@sina.cn (R.C.); patrickyang@zju.edu.cn (B.Y.); 2 Key Laboratory of Agricultural and Animal Products Store and Processing of Guizhou Province, Guiyang 550025, China

**Keywords:** sialic acid, density functional theory, activity index, independent gradient model, solvent effects

## Abstract

The animal product hazard factor *N*-glycolylneuraminic (Neu5Gc) and brain nutrient substance *N*-acetylneuraminic acid (Neu5Ac) were studied at the M062X/6-311 + G(d,p) geometry optimization level. We considered the electronic structure parameters with different solvents: (benzene ε = 2.27, acetic acid ε = 6.25, ethanol ε = 24.85, lactic acid ε = 22.00, formic acid ε = 51.1, water ε = 78.35). The maximum molecular surface electrostatic potentials, which were 62.77 for Neu5Gc and 60.90 kcal/mol for Neu5Ac, are both located on the carboxyl group hydrogen. The orbital analysis showed that the amide group and carboxyl group confer the sites with susceptibility to nucleophilic and electrophilic attack, respectively. The solvent effect showed that polar solvents, such as formic acid and water, can enhance the two molecules’ nucleophilic activity. To better understand the roles of the hydroxyl group in the two molecules, the independent gradient model theory confirmed the four intramolecular hydrogen bonds of Neu5Gc at gas phase, whereas Neu5Ac only has two. The lowest bond dissociation energy in solvent occurs at O7-H, which is 104.03 kcal/mol in water for Neu5Gc and 104.57 kcal/mol in lactic acid for Neu5Ac. The lowest proton affinity value for Neu5Gc (20.34 kcal/mol) and Neu5Ac (20.76 kcal/mol) was both occur at the carboxyl group O6-H under ethanol. The antioxidant mechanisms of the two sialic acid are prone to sequential proton-loss electron transfer under polar or non-polar solvents.

## 1. Introduction

Sialic acids are a series of nine-carbon backbone acid amino-group sugars, systematically called 5-amino-3,5-dideoxy-d-glycerol-d-galactosidoses [1]. In animals, 99% of sialic acid exists in the forms of *N*-acetylneuraminic acid (Neu5Ac) and *N*-glycolylneuraminic acid (Neu5Gc). Sialic acids play an important role in the physiological processes of the human body, such as cell recognition, tumor metastasis, and immune system regulation [2]. Many studies have showed that Neu5Gc cannot be detected in normal human tissues, but sialic acid is abundant in other mammals. This is due to a deletion within the human gene responsible for converting the *N*-acetyl to the *N*-glycolyl form, CMP-*N*-acetylneuraminic acid hydroxylase (CMAH). The only sialic acid found in normal humans is Neu5Ac, which is a nutrient substance, abundant in brain nerve tissues. Generally, Neu5Gc accumulates in the human body through cancerous tissue and the consumption of red meat (beef, pork, lamb, etc.) [3,4]. On October 26, 2015, the International Agency for Research on Cancer (IARC) monographs program classified the consumption of red meat as probably carcinogenic to humans (Group 2A), thus making Neu5Gc a hazard factor [5,6].

In food processing, the physical methods (frying, cooking, etc.) and biochemical methods (fermentation, enzymes, etc.) used do not reduce Neu5Gc in foods. The molecule’s reactivity is related to its structural characteristics [7], and studies on sialic acid are mainly focused on its conformations, and its interactions with biomolecules. Veluraja et al. studied the dominant conformation of sialylated oligosaccharides and their binding to sialidase at the semi-empirical calculation level [8]. van Lenthe et al. used ab-initio calculation theoretical level HF/6-31G(d) to study the charge distribution and orbital information of six sialic acid derivatives [9]. Priyadarzini et al. studied the conformation and inter-molecular hydrogen bonding of Neu5Ac in a biological environment via molecular dynamics simulations and quantum chemical calculations [10]. Sawada et al. determined the optimized geometry of Neu5Ac and the inter-molecular hydrogen bond between water molecules at the B3LYP/6-31G(d,p) theoretical level [11]. Blessy et al. examined molecular docking, and molecular dynamics simulation revealed the key residue information of the Neu5Gc interaction with cholera toxin [12]. However, studies on the physicochemical properties of sialic acid molecules are rare and the effect of food processing solvents on the sialic acid reactivity is unknown.

Sialic acids can be released freely into the cell tissue and react with a large amount of free radicals that are generated in the human body during metabolic processes [13]. The scavenging activity of some monosaccharides for hydroxyl radicals has been successfully proven [14,15]. 1-Kestose, 6-kestose, and nystose have shown their capacity to donate electrons when reacting with free radicals. Neu5Ac and Neu5Gc are all pyranose derivatives that also have certain antioxidant activity. Iijima et al. found that Neu5Ac is a potential antioxidant that can reduce the cytotoxicity of lipid hydroperoxides by direct reaction with hydrogen peroxide [16]. This activity can counteract the cytotoxicity of hydrogen peroxide (H_2_O_2_), and the anti-toxicity is the result of a straight chemical reaction [17]. Serdar et al. found that total sialic acid is related to lipid and protein oxidation markers [18]. Sialic acids can occupy the outermost termini of many *N*-linked and *O*-linked glycoprotein glycans in mammals [19]. Reuter et al. proved that bound sialic acid also has a certain antioxidant capacity [20]. Ogasawara et al. revealed that mucin acts as a hydroxyl radical scavenger via a direct reaction with its connected sialic acids [21]. However, the antioxidant mechanisms of sialic acid under different solvents are not clear. Based on these unknowns, we have studied the molecular electronic structure, reactivity, and antioxidant mechanism of Neu5Gc and Neu5Ac using quantum chemical calculations in order to provide reference information for the applications of sialic acids in the medicine, food, and health areas. 

## 2. Computational Details

### 2.1. Molecular Structure Parameters Calculation

The model molecular structures of Neu5Ac and Neu5Gc were taken from the PubChem database [22]. Geometry optimization and vibration frequencies analysis of Neu5Gc and Neu5Ac were calculated at the M062X/6-311 + G(d,p) level under gas phase conditions and in six solvent environments (ε = dielectric constant): benzene (ε = 2.27), acetic acid (ε = 6.25), ethanol (ε = 24.85), lactic acid (ε = 22.00), formic acid (ε = 51.1), and water (ε = 78.35). The optimized geometries of all minima of Neu5Gc and Neu5Ac are characterized by the absence of imaginary frequencies. The presence of a solvent means that a molecular solvent can interact with the solute molecular and form a shell around the solute with different thicknesses. As the solubility, dielectric constant, and polarity of solvent can affect the structure and charge distribution of a molecule, the solvent effect was calculated using the continuum solvation model density (SMD) [23]. The lactic acid solvent parameters were fitted by Gaussian 16 manually-defined solvents for SMD calculations.

The molecular electrostatic potential (ESP) at van der Waals (vdW) surface, frontier molecular orbital (FMO), natural bond orbital (NBO), and the conceptual density functional (CDFT) activity index were calculated based on the optimized structure at the M062X/6-311G(d,p) level. CDFT activity is related to chemical reactivity descriptors of chemical compounds, which include local properties (Fukui functions) and global properties (electronegativity χ, chemical potential μ, chemical hardness η, chemical softness s, and electrophilic index ω) [24]. The molecular chemical potential values are equivalent to the element electronegativity when calculated using density functional theory; hence, the global descriptors can be approximated by the highest occupied molecular orbital (HOMO) and the lowest unoccupied molecular orbital (LUMO). The calculation formula is as follows:

Electronegativity: χ = −1/2 (ELUMO + EHOMO)(1)

Chemical potential: μ= −χ(2)

Electrophilic index: ω = μ^2^/2η (3)

Chemical hardness: η = 1/2 (ELUMO − EHOMO)(4)

Chemical softness: s = 1/(2η)(5)

The Fukui function represents the changes in the electron density of a point (atom or functional group) with the number of electrons. The condensed Fukui function (CFF) functions were obtained by approximate integrations of the Fukui function over atomic regions, which enables the quantitative analysis of atomic charge. If the orbital relaxation effect is not significant, the Fukui function can be rationalized from frontier molecular orbital theory using Koopmans’ theorem [25]. Function qA was defined to represent the Hirshfeld charge of atom A, then CFF can be calculated by the following equations:

(6) $$\mathrm{Governing}\;\mathrm{electrophilic}\;\mathrm{attack}:\;f_{A}^{-}=q_{N-1}^{A}-q_{N}^{A}$$

(7) $$\mathrm{Governing}\;\mathrm{nucleophilic}\;\mathrm{attack}:\;f_{A}^{+}=q_{N}^{A}-q_{N+1}^{A}$$

(8) $$\mathrm{Governing}\;\mathrm{radical}\;\mathrm{attack}:\;f_{A}^{0}=\left(q_{N-1}^{A}-q_{N+1}^{A}\right)/2$$

where *q_N_*, *q_N_*_+1_, and *q_N_*_−1_ represent the electron density of the system in the initial state, ionization of one electron and the state of combining one electron, respectively [25]. 

The Hohenberg-Kohn theorem confirmed that the density ρ is the fundamental property that characterizes the ground state of a system. Atoms in Molecules (AIM) states that the electron density distribution is related to the chemical bonding type [26]. The bond critical point (BCP) occurs between attractive atom pairs. The Laplacian (∇^2^ρ) plays an important role in the characterization of chemical bonding. The relationships between energetic topological parameters and the ∇^2^ρ(r) at BCPs is:

(9) $$\frac{1}{4}\nabla^{2}\rho (r)=2G\;(r)\;+\;V\;(r)$$

where G(r), V(r), and ρ(r) are the kinetic energy, potential energy, and the total electron energy densities respectively. For a closed shell system, a positive ∇^2^ρ indicates a hydrogen bond. The bond energy E_HB_ can be described as E_HB_ = 1/2 V (r_BCP_) in kcal/mol.

### 2.2. Antioxidant Mechanism 

The antioxidant process can be thought of as the transfer of hydrogen atoms and electrons during interaction with free radicals. Since the antioxidant mechanisms of Neu5Ac and Neu5Gc are not clear, the energy values of the four common antioxidant pathways were calculated separately [27,28].

The hydrogen atom transfer mechanism (HAT) is calculated as:

ROH + X^•^ → RO^•^ + XH (10)

This is the hydrogen extraction process, where the radical X^•^ transfers the H in the organic molecule to itself and causes the organic molecule to become a radical. The bond dissociation energy (BDE) value is related to this process: the lower the BDE value, the better the antioxidant activity. 

Single electron transfer mechanism (SET) occurs as:

ROH + X^•^ → ROH^•+^ + X^−^ (11)

The ionization potential (IP), also called the electron transfer enthalpy is related to this process. The lower the IP value, the easier the electrons are extracted.

Single-electron transfer followed by proton transfer mechanism (SET-PT) occurs as:

ROH + X^•^ → ROH^•+^ + X^−^ (12)

ROH^•+^ → RO^•^ + H^+^ (13)

The first step is related to IP, and the second step is related to the proton Dissociation Enthalpy (PDE). The sequential proton loss electron transfer mechanism (SPLET) is:

ROH → RO^−^ + H^+^ (14)

RO^−^ + X^•^ + H^+^ → RO^•^ + XH (15)

The intermediate product of ROH forms free radicals RO^•^ with the free radicals X^•^. This step commonly occurs in polar environments. The first step is related to the proton affinity (PA), and the second step is related to the electron Transfer enthalpy (ETE). The calculation formula above is listed here:

IP = H(ROH^•+^) + H(e^−^) − H(ROH) (16)

BDE = H(RO^•^) + H(H^•^) − H(ROH) (17)

PDE = H(RO) + H(H+) − H(ROH^•+^) (18)

PA = H(RO^−^) + H(H^+^) − H(ROH) (19)

ETE = H(RO^•^) + H(e^−^) − H(RO^−^) (20)

The equations enable the calculation of the enthalpy of the parent molecule H(ROH), its radical cation H(ROH^•+^), radical H(R^•^), and anion H(RO^−^). The thermodynamic quantities for the gas-phase include the commonly accepted values of −312.30 kcal/mol for the hydrogen atom, 0.75 kcal/mol for electron enthalpy, and 1.48 kcal/mol for proton enthalpy. The reference values of the enthalpy of the hydrogen atom, electron, and proton at the studied solvent conditions were referenced from the literature [29,30]. Enthalpy was the energy item used for the study of the antioxidant mechanism, which was calculated by the sum of the high precision single point energy at the M062X/def2TZVPPD level and the thermal correction to enthalpy at the M062X/6-311 + G(d,p) level. The thermal correction of enthalpy was determined using the scale factors 0.97 at zero point vibration energies [31]. 

All the quantum chemical calculations were performed using the Gaussian 16 (Revision A. 03) package supported by the Guizhou University computing cluster [32]. ESP, FMO, CDFT reactivity index calculations, and the intramolecular hydrogen bonds topological analyses of Neu5Ac and Neu5Gc were performed by Multiwfn 3.6 suite on a personal computer [33]. Among them, the ESP on the vdW surface in the Multiwfn program set was referenced in Lu’s work as follows: the grid spacings were set to 0.2 Bohr and the vdW surface denotes the iso-surface of ρ = 0.001 e/bohr3 [34]. All the iso-surface maps were generated with the VMD 1.9.1 program [35].

## 3. Results and Discussion

### 3.1. Molecular Structure and Electrostatic Potential

**Figure 1:**
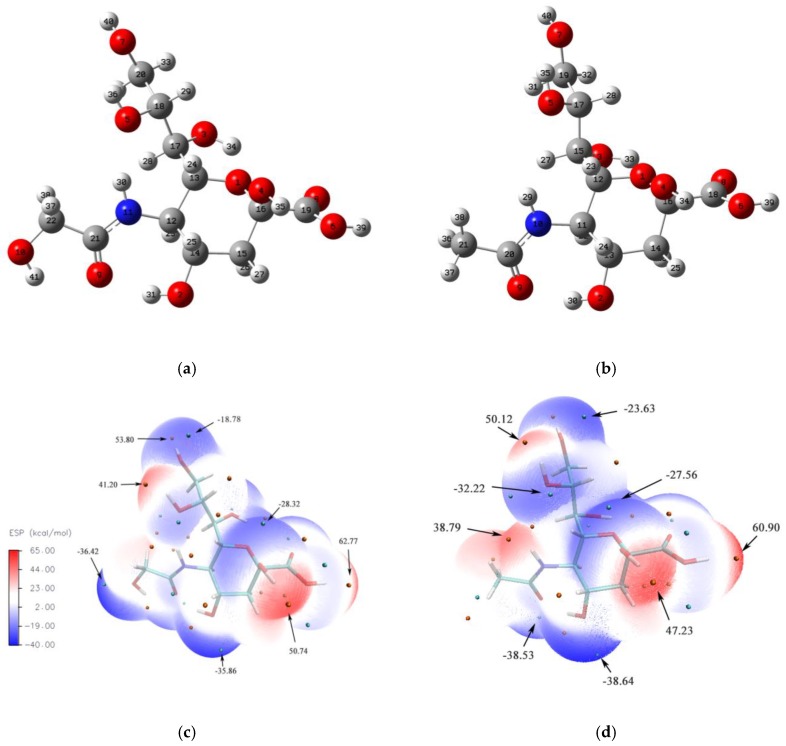
The gas phase optimized structure and molecular electrostatic potential (ESP) of Neu5Gc and Neu5Ac. Note: (**a**) The optimized structure of Neu5Gc, (**b**) The optimized structure of Neu5Ac, (**c**) The ESP of Neu5Gc, (**d**) The ESP of Neu5Ac. Unit: kcal/mol.

Figure 1 shows the optimized molecular structure and ESP of Neu5Gc and Neu5Ac in the gas phase. For Neu5Gc, the length of bonds C22–C21, N11–C12, and C17–C18 related to the pyran ring side chain are 1.521, 1.462, and 1.524 Ǻ long, respectively. At the same position in Neu5Ac, the bond lengths are 1.509, 1.461, and 1.525 Ǻ, respectively. Due to the electron withdrawing effect of hydroxy (O10-H41) at the Neu5Gc hydroxyacetyl group, the C22–C21 bond in Neu5Gc was somewhat longer than C21–C20 in Neu5Ac. 

When comparing the bond distances of bonds (C12–C13, C12–C14, O1–C13, and O1–C16) that most contributed to the two molecule’s skeletons, the distances in Neu5Gc (1.535, 1.533, 1.416, and 1.390 Ǻ, respectively) approach the corresponding positions in Neu5Ac (1.534, 1.532, 1.420, 1.387 Ǻ, respectively). The angle of carboxyl O–C=O in Neu5Gc (124.3°) was similar to that of Neu5Ac (124.1°). However, as the hydroxyl group has the ability to have a donor electronic effect on the Neu5Gc hydroxy acetyl group, the acetyl C–N=O angle (123.8°) in Neu5Gc is little greater than in the Neu5Ac values (122.0°) [36]. The other lengths and angles in the molecule’s framework were also calculated (data not shown, Table S1).

ESP provides a three-dimensional (3D) visualization of electron density distribution and is a tool for predicting favorable sites for electrostatically-dominated non-covalent interactions [37]. From Figure 1, the blue regions gathered with negative charges indicate a vulnerable site for electrophilic attack, and the red regions with positive charge are sites for nucleophilic attack. The lone pair oxygen connected to the hydroxyl group and the amide group mainly occupies the most negative areas, and leads to a minimum value of −36.42 kcal/mol and −38.64 kcal/mol for Neu5Gc and Neu5Ac at vdW surfaces, respectively. This indicates that the hydrogen atom related to the hydroxyl group also may have nucleophilic activity. The maximum site is concentrated around the carboxyl hydrogen atom, with value of 62.77 kcal/mol for Neu5Gc and 60.90 kcal/mol for Neu5Ac, which can easily be attacked by free radicals. This finding is consistent with the results for coumaric acids with reactive oxygen species studies [38]. 

### 3.2. Frontier Molecular Orbital and Natural Bond Orbital

**Figure 2:**
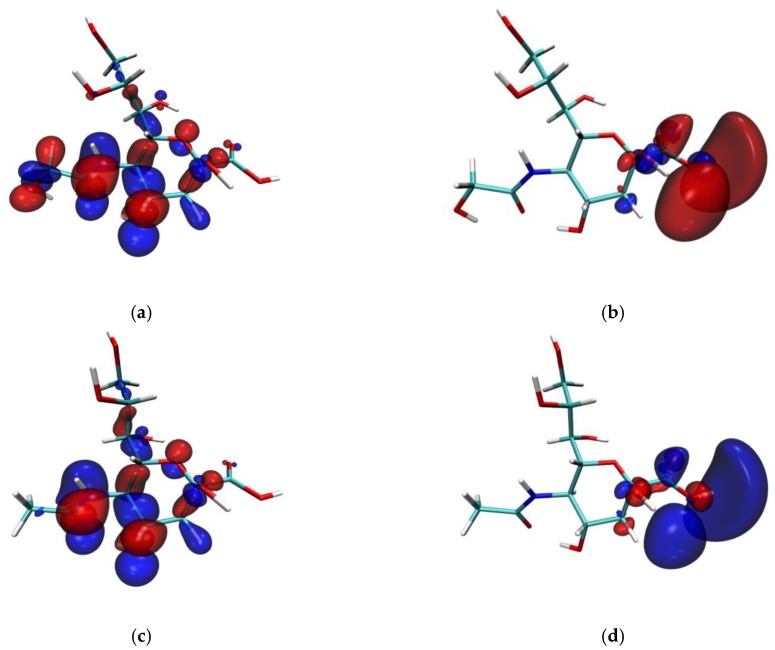
The highest occupied molecular orbital (HOMO) and the lowest unoccupied molecular orbital of Neu5Gc and Neu5Ac. Note: (**a**) The HOMO of Neu5Gc; (**b**) The LUMO of Neu5Gc; (**c**) The HOMO of Neu5Ac; (**d**) The LUMO of Neu5Ac.

The frontier molecular orbital acts as an indicator of molecular reactivity. The molecule orbitals iso-surface map of the two sialic acid molecules is shown in Figure 2. Based on the frontier molecular orbital theory, EHOMO characterizes the ability to donate electrons. The larger the EHOMO, the stronger the electron donation ability. ELUMO indicates the ability of a molecule to attract electrons. The lower the ELUMO, the weaker the electron attraction ability. In Figure 2, the red and blue represent the positive and negative phases of the orbit, respectively. From the plot, the HOMO values of Neu5Gc and Neu5Ac are mainly distributed among the pyranose ring, amide group, and hydroxyl groups, which are susceptible to attacked by electrophiles. The LUMO of the two molecules are also mainly distributed at the carboxyl group, which is an area susceptible to attack by nucleophiles. The nucleophilic electrophilic activity is consistent with the ESP analysis, and aligns with the reported orbital studies on collagen amino acids [39]. The HOMO at the end of the two molecules’ acetyl groups showed little difference. No HOMO was distributed at the Neu5Ac terminal methyl (C21-H36-H37-H38), which maybe one of the reasons for the difference in the activity of the two molecules. The molecular NBO is associated with the nature of intramolecular and intermolecular interactions, charge transfer, and covalent bonding. From the FMO analysis results, the amide, carboxyl, and hydroxyl groups are related to the reactive activity of the two molecules. In order to understand the various second-order interactions between the filled orbital and vacant orbital, especially for the amide groups and carboxyl groups of Neu5Gc and Neu5Ac, we applied NBO methods to mainly discuss the transition mode and interaction of related bonds. The strength of the interaction (stabilization energy) E(2) associated with the electron delocalization i→j was estimated as:

(21) $$E(2)=\;\Delta E_{\mathrm{ij}}=q_{i}\frac{F_{\left(i,j\right)}^{2}}{\varepsilon_{j}-\varepsilon_{i}}$$

where i represents the donor, j represents the acceptor, q_i_ is the orbital occupancy, ε_i_ and ε_j_ are diagonal elements, and F(i,j) is the off-diagonal Fock matrix element. The larger the stabilization energy E(2) value, the more intensive interaction between electron donors and electron-acceptors [40]. The intramolecular interactions of Neu5Gc and Neu5Ac are mainly based on the transition of the anti-bond orbits σ* (C-C), σ* (C-H), σ* (N-C), σ* (N-H), σ* (O-C), σ*(O-H), and π* (O-C) between the bond orbits σ (C-C), σ (C-H), σ (N-C), σ (N-H), σ (O-C), σ (O-H), and π (O-C). For Neu5Gc, two larger orbitals transition occur: one is produced by the electron transfer at the amide group, C22-H37→O9-C21 (σ→π*, 5.85 kcal/mol) and N11-H30→O9-C21 (σ→σ*, 5.86 kcal/mol), and the other is C15-H26→O4-C16 (σ→σ*, 5.84 kcal/mol), which forms due to the electron withdrawing effect of the carboxyl group. The lowest stabilizing energy in Neu5Gc occurs at the transition between the amide group and the adjacent carbocyclic ring, N11-H30→C12-C13 (σ→σ*, 0.50 kcal/mol). For Neu5Ac, a larger orbitals transition is produced by the electron transfer from the methyl group to adjacent amide groups and the methylene group on the ring to the adjacent hydroxyl, which denotes C21-H36→O9-C20 (σ→π*, 5.43 kcal/mol) and C14-H26→O4-C16 (σ→σ*, 5.75 kcal/mol), respectively. The transition with the lowest stabilizing energy of Neu5Ac occurs on the alkane chains, such as, C11-H22→C11-C12 (σ→σ*, 0.52 kcal/mol) and C15-C17→C15-H27 (σ→σ*, 0.52 kcal/mol). From the above, the interaction strengths for Neu5Gc at the amide groups and carboxyl groups are somewhat highger than those for Neu5Ac, which can be one reason for the differences in ESP. The other bond transitions was fully calculated. (data not shown, Table S2.) 

### 3.3. Intramolecular Hydrogen Bonds

Intramolecular hydrogen bonds impact the melting point, conformation stability, acidity, and other properties of a molecule. Hydrogen bonds occur via electrostatic interactions. A hydrogen atom bonds to two electronegative atoms (nitrogen, oxygen, fluorine, etc.), one of which is the hydrogen-bond donor thant has a stronger bond between itself and the hydrogen. We completed a BCP search and intramolecular hydrogen-bond topological analysis of Neu5Ac and Neu5Gc at three different polar conditions (Table 1). From Table 1, the hydrogen bonds of the two sialic acid molecules are O-H...O and N-H...O. Neu5Gc forms more intramolecular hydrogen bonds than Neu5Ac in the gas phase, which may be because Neu5Gc has one more hydroxyl group than Neu5Ac. 

The hydrogen bonding qualitative standards were ρ (0.002–0.04 a.u) and ∇^2^ρ (0.02–0.15 a.u) [41]. Based on the study of Rozas et al., the strength of hydrogen-bonds can be classified into three types: weak hydrogen bonds (E_HB_ < 12.0 kcal/mol, ∇^2^(r_BCP_) > 0, and G(r_BCP_) + V (r_BCP_) > 0), medium hydrogen bonds (12.0 < E_HB_ < 24.0 kcal/mol, ∇^2^(r_BCP_) > 0, and G(r_BCP_) + V (r_BCP_) < 0), strong hydrogen bonds (E_HB_> 24.0 kcal/ mol, ∇^2^ (r_BCP_) < 0, G(r_BCP_) + V (r_BCP_) < 0) [42]. According to the classification criteria above, the hydrogen bonds of Neu5Gc and Neu5Ac that form are all weak hydrogen bonds (E_HB_ < 12.0 kcal/mol, ∇^2^(r_BCP_) > 0, G(r_BCP_) + V (r_BCP_) > 0). The strongest hydrogen bond of Neu5Gc is N11-H30...O5 in the gas phase, and E_HB_ is −23.2997 kcal/mol; the strongest hydrogen bond of Neu5Ac is O3-H33...O1 in the benzene phase, and the E_HB_ is −24.02 kcal/mol. When comparing the topological parameters of hydrogen bond O2-H...O9 of the two molecules in the gas phase, the hydrogen bond strength of Neu5Gc is weaker than that of Neu5Ac. This maybe induced by the electron density difference. The decrease and increase in the electron density at O9 may be caused by the methyl electron-withdrawing effect at the Neu5Ac amide group and the electron donating effect of hydroxyl connected with amide group at the Neu5Gc, respectively.

### 3.4. IGM Analysis of Intramolecular Hydrogen Bonds

**Figure 3:**
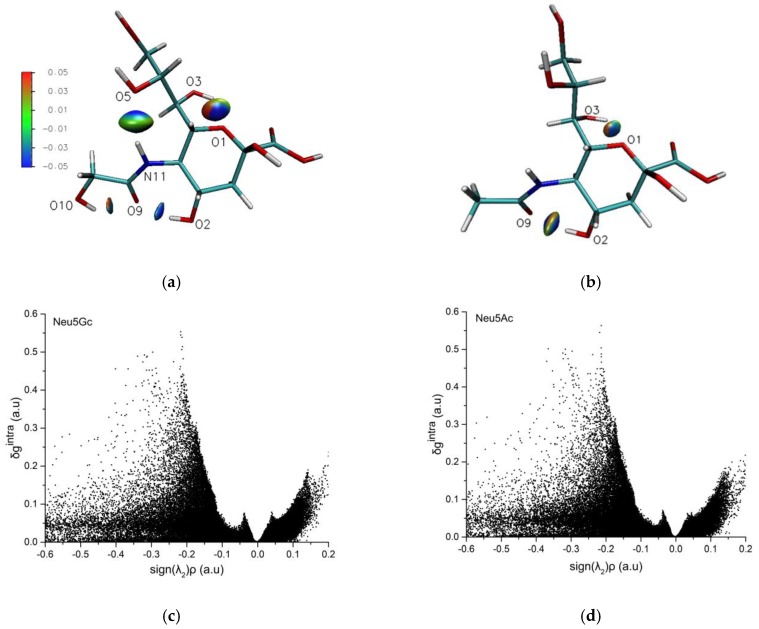
The independent gradient model (IGM) iso-surface and scatter plot of Neu5Gc and Neu5Ac. Note: (**a**) The iso-surface of Neu5Gc intramolecular non-covalent interaction; (**b**) The iso-surface of Neu5Ac intramolecular non-covalent interaction; (**c**) The scatter plot of Neu5Gc intramolecular non-covalent interaction; (**d**) The scatter plot of Neu5Ac intramolecular non-covalent interaction.

The reduced density gradient function is a method to analyze non-covalent interactions based on the electron density topology. The function enables the identification and visualization of weak interactions in 3D real space in the form of chemically intuitive iso-surfaces. Defining the iso-surfaces as function S, S can be described as [43]:

(22) $$S=\frac{1}{2{\left(3\pi^{2}\right)}^{1/3}}\frac{\left|\nabla \rho (r)\right|}{{\left(\rho (r)\right)}^{4/3}}$$

where ρ(r) represents the electron density (ED) and |∇ρ(r)| is the norm of the ED gradient vector. The basis of the IGM is the ED gradient in terms of atomic components. As S^IGM^ is based on the |∇ρ(r) ^IGM^|, the largest deviations of S^IGM^ and S occur at the BCP. Set the difference as function δg, δg(r) = |∇ρ(r)^IGM^| − |∇ρ(r)|. δg can divided into intramolecular interaction (δg^intra^) and intermolecular interaction (δg^inter^). By projecting the δg descriptor with the sign function sign(λ^2^)ρ, the scatter plot was obtained The non-covalent interaction of Neu5Gc and Neu5Ac via IGM was determined based on the optimized geometry in the gas phase. The iso-surface and scatter plot results are shown in Figure 3.

In the iso-surface map (Figure 3a,b), the deep-blue color regions indicate stronger electrostatic attraction, and the green color regions represent weak van der waals interactions. It can be seen from the isosurface map that there are four intramolecular hydrogen bonds in Neu5Gc, O2-H31...O9, O3-H34...O1, O10-H41...O9, and N11-H30...O5, and only two O3-H33...O1 and O2-H30...O9 in Neu5Ac, this is consistent with the topological analysis findings.

In the scatter plots (Figure 3c,d), the default range of sign(λ^2^) ρ is from −0.05 to 0.05. According to the IGM theory, for our system, when sign(λ^2^) ρ nears −0.04, a peak in the δginter polt occurs, which represents the intermolecular interaction hydrogen bond. When sign(λ^2^) ρ nears −0.2, a larger peak in the δg^intra^ plot occurs, which represents the intramolecular interaction, and positive sign(λ^2^) ρ values represents spatial repulsion. From Figure 3, in the region of −0.2 for both Neu5Gc and Neu5Ac, the characteristic peaks of −0.2 indicate very weak intramolecular hydrogen bonds.

### 3.5. Molecular Reactivity Index

The molecular reactivity index (HOMO, LUMO, Egap, hardness, softness, electronegativity, chemical potential, and electrophilic index) of Neu5Gc and NeuAc was calculated as shown in Table 2. Table 2 shows that the smallest HOMO and LUMO values of Neu5Ac Neu5Gc occur under gas. For Neu5Gc, the lowest Egap occurs in the gas phase, indicating that the reactivity under gas is higher than with solvents. In solvent media, the lowest Egap is 9.699 eV in ethanol, and the highest Egap is 9.840 eV in lactic acid, showing that the activity under ethanol is higher than under lactic acid. This may be related to the proton solvent ethanol that can generate more intermolecular hydrogen bonds with Neu5Gc, indirectly reducing the transition energy [44]. 

Hard-Soft-Acid-Base (HSAB) theory treats electrophilic reagents lacking electrons as Lewis acids, and electron-rich substrates as Lewis bases, and Hard (soft) acids prefer to react with hard (soft) bases. The hardness of Neu5Gc under solvents is totally greater than in gas conditions. The largest is under lactic acid. the smallest is under ethanol. The smallest electronegativity and electrophilic index of Neu5Gc are also under lactic acid. The food fermentation process provides an ideal solvent environment with lactic acid and ethanol. According to the HASB principle, Neu5Gc tends to react with lactic acid and ethanol. This is consisting with Chen’s liquid chromatography-mass spectrometry (LC-MS) detection studies, which showed that the content of Neu5Gc in fermented foods significantly lower than in other processing products [45]. 

For Neu5Ac, the lowest Egap value is 9.578 eV under formic acid. The least hardness occurs under formic acid as well. Therefore, Neu5Ac can act as a soft Lewis acid to react easily with lactic acid. In the water phase, the hardness, electronegativity, and electrophilic index of Neu5Ac have the largest values, but the softness and chemical potential are the smallest, verifying that Neu5Ac easily captures electrons in polar water solvents.

### 3.6. Molecular Condensed Fukui Function

From the ESP, FMO, BCP, and IGM results above, the role of the hydroxyl group in the two molecules can not be neglected. In order to quantitatively analyze the reactive activity, the condensed Fukui functions of the hydroxyl oxygen atom of Neu5Ac and Neu5Gc were calculated based on Hirshfeld charges. Here, we only discuss the nucleophilic and electrophilic activities under different solvents. The results are listed in Table 3 and Table 4. 

The nucleophilic activity of the Neu5Ac oxygen atoms O5, O6, O4, and O3 is increased sequentially under formic acid, water, and ethanol. The nucleophilic activity of the Neu5Ac hydroxy oxygen atoms (O2, O3, O4, O6, and O7) follows the order water < acetic acid < benzene < gas phase. The nucleophilic activity of the Neu5Gc oxygen atoms O7, O6, O3, O10, O9 increases sequentially, and O3, O4, O6, O7 follow the order water < ethanol < benzene < gas phase. O1, O5, and O9 follow: gas phase < benzene < water < formic acid. The nucleophilic activities of O1, O2, O3, O4, O5, O6, and O7 of Neu5Gc are larger than Neu5Ac under water conditions. The electrophilic activity of the oxygen atom of Neu5Ac (O7, O9, O3, O2, O4, and O6) increase sequentially in gas and benzene phase. O7, O3, O2, O4, and O6 increase sequentially in formic acid, ethanol, and water. For Neu5Gc, the electroactive activity of the oxygen atom satisfies the order: O7 < O3 < O2 < O4 < O6 in all studied conditions. O7, O10, O5, O9, O3, O2, O4, and O6 increase sequentially in formic acid, ethanol, and water. The electrophilic activity of O3, O4, O6, and O7 in Neu5Gc is larger than Neu5Ac under benzene.

According to the activity sequence above, we confirmed that the polarity of the solvent has a little effect on the electrophilic activities of a single hydroxyl oxygen atom. The electrophilic activity of the alcohol hydroxyl oxygen atom of the two molecules follows the order water < formic acid < acetic acid < benzene, whereas the carboxyl oxygen atom follows the order gas phase < lactic acid < ethanol. This proves that the polarity of the solvent affects the electrophilic nucleophilic activity of the molecular functional groups [46].

### 3.7. Antioxidation Mechanism of Neu5Ac and Neu5Gc

The CFF results above reveal that the hydroxyl group of the Neu5Gc and Neu5Ac is related to the electrophilic nucleophilic activity, such as that toward free radicals. In order to explore the mechanism of anti-hydrogen peroxide pressure ability of the two sialic acid molecules. we calculated the four possible typical pathways. The BDE, IP, PDE, PA, and ETE values of Neu5Gc and Neu5Ac O-H under gas and six solvents are listed at Table 5, Table 6 and Table 7.

#### 3.7.1. Hydrogen Atom Transfer Mechanism (HAT)

In the HAT mechanism, a hydrogen atom directly transitions from an anti-oxidant to a free radical to break the chain reaction. The BDE values measure the energy required to break down a chemical bond, and are associated with the process. From Table 5 and Table 6, the BDE values for the O-H in Neu5Gc and Neu5Ac are close, and the smallest value occurs at O7-H of Neu5Gc under gas, at 103.51 kcal/mol. The BDE at sites O2-H, O3-H, O4-H, O5-H, and O6-H of Neu5Gc are the lowest in the gas phase, whereas the corresponding positions in Neu5Ac are the smallest in water. Under solvent conditions, the minimums both occur at the O7-H position, which is 104.03 kcal/mol in water for Neu5Gc and 104.57 kcal/mol in lactic acid for Neu5Ac. This indicates that the O7-H site is most susceptible to nucleophilic attack, which is consistent with our finding from the condensed Fukui function analysis. Referencing the BDE of gallic acid (79.79 kcal/mol) and resveratrol (82.50 kcal/mol), the two molecules have a certain antioxidant activity [47]. 

For Neu5Gc, the BDE of O4-H, O5-H, O3-H, and O6-H increase sequentially; O3 and O5 follow the order water < lactic acid < gas phase < benzene; and O2, O4, O6, and O7 are ordered as gas phase < lactic acid < ethanol < acetic acid. For Neu5Ac, the BDE of O4, O5, and O6 increase sequentially; O3 and O5 follow the order water < ethanol < benzene < acetic acid; and O2, O4, O6, and O7 are ranked in the order gas phase < benzene < ethanol < formic acid. This shows that the nucleophilic activity of O2, O4, O6, and O7 improved in polar solvents, which is consistent with the CFF results. Under non-polar solvent conditions (benzene), the lowest BDE value was at the O2-H of Neu5Gc (104.78 kcal/mol) and O7-H of Neu5Ac (104.85 kcal/mol). 

This indicates that the O2-H, O4-H, O6-H, and O7-H BDE values of the two molecules in polar environments are larger than non-polar conditions, revealing that these sites are susceptible to nucleophilic attack under polar solvents. This phenomenon may be caused by the reactivity of the nucleophiles increasing with the solvent’s dielectric constant values [48].

#### 3.7.2. Single Electron Transfer Mechanism (SET)

The IP values are related to SET. Table 7 shows that the IP values of Neu5Ac and Neu5Gc are significantly larger than the BDE value and that the single electron transfer mechanism was not preferred over the hydrogen atom transfer mechanism when sialic acid scavenges free radicals. The IP values of Neu5Gc were always larger than those of Neu5Ac under the studied conditions, and satisfied the order: water < formic acid < acetic acid < lactic acid < ethanol < benzene < gas phase. So, electrons are easily lost and the electrophilic capacity increases with polar solvents due to the radical cations being charged and quite sensitive to the polarity of the solvents [49]. Among them, the lowest IP value was 131.63 kcal/mol for Neu5Gc and 131.99 kcal/mol for Neu5Ac under water, indicating that the Neu5Ac more easily extracts the electrons and experiences nucleophilic attack in water. This is consistent with the CDFT results, which showed that the electrophilic index of Neu5Gc is larger than Neu5Ac under water. 

#### 3.7.3. Single-electron Transfer Followed by Proton Transfer Mechanism (SET-PT)

The PDE values can determine the second step of SET-PT and demonstrate that the thermodynamics preferred the hydroxyl group to deprotonation to form a radical cation. Table 5 and Table 6 show that the PDE values of Neu5Gc and Neu5Ac are the largest in the gas phase and significantly larger than the corresponding BDE and IP values. This illustrates that the proton loss process does not easily occur after the first step of electron loss in the gas phase. Compared with non-polar solvent conditions, the PDE values with polar solvents are smaller than the BDE and IP values. The lowest PDE values of Neu5Gc (5.65 kcal/mol) and Neu5Ac (7.35 kcal/mol) occur both at the O7-H position under ethanol, which is consistent with the order of the BDE values. For solvent polarity, the PDE values of Neu5Gc and Neu5Ac follow: ethanol < carboxylic acid < lactic acid < benzene < gas phase. Under polar solvents, the PDE values of O2, O3, O4, and O6 in Neu5Gc are smaller than the Neu5Ac positions. This indicates that Neu5Gc is more prone to proton transfer after the single electron transfer occurs in polar solvents than Neu5Ac.

#### 3.7.4. Sequential Proton Loss Electron Transfer Mechanism (SPLET)

Table 5 and Table 6 show that the PA values of Neu5Gc and Neu5Ac satisfy the order PA < BDE < IP, and the values in the gas phase are obviously larger than in solvent conditions, and much smaller in the polar solvents than in the non-polar solvent’s conditions. The lowest PA values for Neu5Gc (20.34 kcal/mol) and Neu5Ac (20.76 kcal/mol) are both at the carboxyl O6-H under ethanol, which is consistent with PDE values. Under non-polar solvent conditions (benzene), the lowest PA values of Neu5Gc (80.55 kcal/mol) and Neu5Ac (81.95 kcal/mol) are both at O6-H, which is the same as in gas phase conditions, being consistent with the LUMO map results. This indicates that when compared with the HAT and the SET mechanism, the first step of the SPLET mechanism (proton loss) is thermodynamically favorable under polar solvent [50]. From Table 3, the ETE values of the two molecules in polar solvents are less than in the non-polar solvent, which is verified by the single electron transfer result. The ETE values of the alcoholic hydroxyl group follow: PA < ETE < BDE < IP, showing that electron transfer after the proton loss step is thermodynamically allowed to proceed. Under the polar solvents, the lowest ETE value of Neu5Gc (79.67 kcal/mol) was at O10-H under acetic acid, whereas that for Neu5Ac was at O3-H position (80.77 kcal/mol), with a difference of only 0.1kcal/mol. Under the non-polar solvents (benzene), the lowest ETE value occured at O10-H of Neu5Gc (77.65 kcal/mol) and at O3-H of Neu5Ac (79.96 kcal/mol). Hence, after proton loss, the antioxidant activities of the two molecules are very similar. When compared with the lowest BDE, IP, PA, PDE, and ETE valeus with different solvents, we inferred that when Neu5Gc and Neu5Ac scavenge free radicals under solvents, the proton loss of the carboxyl O6-H is the first step in the SPLET mechanism, which then continues with electron transfer of the other alcoholic hydroxyl hydrogen atoms. 

## 4. Conclusions

In this work, the molecular electronic structure properties, molecular activity index, and antioxidant mechanism of Neu5Gc and Neu5Ac in different solvents were studied using density functional theory calculations. The molecular surface electrostatic potential shows that the positive charges are concentrated nearby the carboxyl group, with maximum values of 62.77 for Neu5Gc and 60.90 kcal/mol for Neu5Ac, mainly distributed in the LUMO orbital and with high nucleophilic activity. The negative charges are concentrated near the amide group, with the minimum values of –36.42 for Neu5Gc and −38.53 kcal/mol for Neu5Ac, mainly distributed in the HOMO orbital and with high electrophilic activity. The lowest Egap value of Neu5Ac is 9.5787 eV under formic acid, and 9.699 eV for Neu5Gc with ethanol. Natural bond orbital analysis revealed that the intramolecular interactions of Neu5Gc and Neu5Ac are mainly caused by transitions between the anti-bond orbit σ* and the bonding orbit σ. The larger orbital transitions are mainly produced by electron transfer to the amide group. AIM topology and IGM non-covalent interaction analysis indicated that the intramolecular interactions are weak non-covalent hydrogen bonds, and the hydrogen bonds types are O–H...O and N–H...O. The strongest hydrogen bond of Neu5Gc is N11-H30...O5 in the gas phase, whereas it was O3-H33...O1 in the benzene phase for Neu5Ac. In a solvent environment, the lowest BDE and PA values of Neu5Gc and Neu5Ac are 104.03 and 20.34 kcal/mol and 104.57 and 20.76 kcal/mol, respectively. From the calculated energy values of the four possible antioxidant pathways, the free-radical attack nucleophilic activity of the two molecules can be enhanced with a polar solvent, such as formic acid or water, and mainly undergoes the electron transfer of continuous proton loss (SPLET) mechanism, and the carboxyl group O6-H proton loss is the first thermodynamically favored step. This work provides a reference for the in-depth study of sialic acid application in food processing technology.

## Figures and Tables

**Table 1:** Topological parameters at intramolecular bond critical points of Neu5Gc and Neu5Ac. ρ: Electron density (a.u.); ∇^2^ρ: Laplacian of electron density (a.u.); G: Lagrangian kinetic energy (a.u.); H: Hamiltonian kinetic energy (a.u.); V: Potential energy density (a.u.).

Neu5Gc	Bond	ρ	▽^2^ρ	V	G	H_BCP_	E_HB_	Length	Angle
Gas	O2–H31...O9	0.02953	0.1126	−0.02557	0.02687	0.001301	−33.56	2.794	4.804
	O3–H34...O1	0.02885	0.1327	–0.02784	0.03050	0.002673	−36.55	2.524	6.080
	O10–H41...O9	0.02385	0.1051	–0.02086	0.02357	0.002710	–27.39	2.646	13.65
	N11–H30...O5	0.02255	0.09263	–0.01774	0.02045	0.002705	–23.29	2.921	2.811
Acetic acid	O10–H41...O9	0.2266	0.1019	–0.01975	0.2262	0.002869	–25.94	2.653	4.951
Benzene	O10–H41...O9	0.02319	0.1038	–0.02031	0.02313	0.002817	–26.67	2.652	5.516
Gas	O3–H33...O1	0.02262	0.1058	–0.02053	0.02349	0.002964	–26.95	2.586	8.897
	O2–H30...O9	0.02907	0.1101	–0.02485	0.02619	0.001343	–32.62	2.800	4.906
Acetic acid	O2–H30...O9	0.02361	0.08990	–0.01891	0.02069	0.001782	–24.82	2.850	5.233
Benzene	O3–H33...O1	0.02027	0.09740	–0.01829	0.02132	0.003026	–24.02	2.625	11.40
	O2–H30...O9	0.02818	0.1064	–0.02373	0.02518	0.001443	–31.16	2.816	4.511

**Table 2:** Global reactivity index of Neu5Gc and Neu5Ac (at the M062X/6-311G(d,p) level, Egap: EHOMO-ELUMO, χ: electronegativity, μ: chemical potential, ω: electrophilic index, η: chemical hardness, s: chemical softness, unit: eV).

Neu5Gc								
**Media**	**HOMO**	**LUMO**	**Egap**	**η**	**s**	**χ**	**μ**	**ω**
Gas	−8.900	0.753	9.653	4.826	0.1036	4.074	−4.074	1.719
Benzene	−8.867	0.955	9.822	4.911	0.1018	3.955	−3.955	1.593
Ethanol	−8.660	1.039	9.699	4.849	0.1030	3.810	−3.810	1.496
Acetic acid	−8.728	0.995	9.723	4.861	0.1028	3.866	−3.866	1.537
Lactic acid	−8.715	1.125	9.840	4.920	0.1016	3.795	−3.795	1.463
Formic acid	−8.720	1.013	9.733	4.866	0.1027	3.853	−3.853	1.525
Water	−8.731	0.988	9.720	4.860	0.1028	3.871	−3.871	1.542
**Neu5Ac**								
Gas	−8.766	0.878	9.644	4.822	0.1036	3.944	−3.944	1.613
Benzene	−8.674	1.027	9.702	4.851	0.1030	3.823	−3.823	1.506
Ethanol	−8.594	1.068	9.662	4.831	0.1034	3.762	−3.762	1.465
Acetic acid	−8.701	1.021	9.722	4.860	0.1028	3.840	−3.840	1.516
Lactic acid	−8.578	1.141	9.719	4.859	0.1029	3.719	−3.718	1.423
Formic acid	−8.548	1.030	9.578	4.789	0.1043	3.759	−3.759	1.475
Water	−8.718	1.007	9.726	4.863	0.1028	3.855	−3.855	1.528

**Table 3:** Condensed Fukui function of Neu5Gc at the M062X/6-311G(d,p) level, keep four significant digits. (**f** −: electrophilic activity index, **f** +: nucleophilic activity index.)

f −	Electrophilic Activity of Neu5Gc						
**Media**	**O2**	**O3**	**O4**	**O5**	**O6**	**O7**	**O10**
Gas	0.1237	0.02612	0.01854	−0.003166	0.01067	0.009959	0.05184
Benzene	0.08462	0.01581	0.01468	0.001390	0.008207	0.007863	0.04109
Acetic acid	0.06119	0.01006	0.008661	0.002425	0.005481	0.004638	0.03003
Ethanol	0.03166	0.01348	0.008483	0.004389	0.004433	0.003873	0.02739
Formic acid	0.02883	0.01327	0.008223	0.004598	0.003941	0.003317	0.02415
Lactic acid	0.05031	0.009583	0.007909	0.002491	0.004599	0.003322	0.03325
Water	0.03077	0.01305	0.008335	0.004534	0.003866	0.003205	0.02450
**f +**	**Nucleophilic Activity of Neu5Gc**						
Gas	0.02046	0.01737	0.04551	0.001062	0.0694	0.01091	0.01983
Benzene	0.01696	0.01141	0.05685	0.002364	0.09615	0.003067	0.007161
Acetic acid	0.01556	0.006019	0.04722	0.001904	0.1224	0.001077	0.002703
Ethanol	0.01396	0.004336	0.04283	0.001304	0.1240	0.0006920	0.001186
Formic acid	0.01279	0.003578	0.04548	0.001167	0.1269	0.0005470	0.0008410
Lactic acid	0.01419	0.007517	0.04970	0.001725	0.1086	0.001256	0.001962
Water	0.01274	0.003522	0.04491	0.001134	0.1270	0.0005410	0.0007780

**Table 4:** Condensed Fukui function of Neu5Ac at the M062X/6-311G(d,p) level, keep four significant digits. (**f** −: electrophilic activity index, **f** +: nucleophilic activity index.)

f −	Electrophilic Activity of Neu5Ac					
**Media**	**O2**	**O3**	**O4**	**O5**	**O6**	**O7**
Gas	0.1140	0.02236	0.01776	0.0001650	0.01092	0.01185
Benzene	0.06632	0.01300	0.009534	0.0005200	0.007312	0.007243
Acetic acid	0.05073	0.009047	0.008116	0.001855	0.005053	0.004259
Ethanol	0.02826	0.01184	0.007726	0.003867	0.004107	0.003490
Formic acid	0.05069	0.008504	0.007130	0.003015	0.003647	0.002429
Lactic acid	0.05070	0.009064	0.007035	0.002215	0.004367	0.003040
Water	0.04718	0.008686	0.007317	0.003156	0.003574	0.002371
**f +**	**Nucleophilic Activity of Neu5Ac**					
Gas	0.02053	0.01515	0.05226	0.001968	0.08011	0.006588
Benzene	0.01705	0.01137	0.05745	0.002491	0.09739	0.003006
Acetic acid	0.01432	0.006090	0.04783	0.002124	0.1225	0.001194
Ethanol	0.01363	0.004200	0.04485	0.001290	0.1236	0.0006360
Formic acid	0.01236	0.003406	0.04676	0.001115	0.1266	0.0005200
Lactic acid	0.01399	0.007559	0.05077	0.001706	0.1076	0.001251
Water	0.01237	0.003225	0.04587	0.001147	0.1267	0.0005170

**Table 5:** Antioxidant mechanism parameters of Neu5Gc. (optimized and thermal correction to enthalpy at the M062X/6-311 + G(d,p) level, single point energy at the M062X/def2TZVPPD level, kcal/mol).

HAT	BDE of Neu5Gc						
Bond	O2-H	O3-H	O4-H	O5-H	O6-H	O7-H	O10-H
Gas	104.78	108.41	104.14	108.24	114.22	103.51	108.98
Benzene	104.78	109.22	105.93	108.81	115.21	104.79	109.30
Acetic acid	108.56	109.89	108.24	109.72	118.61	106.76	109.51
Ethanol	107.86	108.75	108.10	108.36	117.94	106.04	107.98
Formic acid	109.37	109.86	110.17	110.40	116.21	108.08	109.40
Lactic acid	106.73	107.93	105.98	107.60	114.60	104.55	107.64
Water	105.77	106.59	106.16	106.09	118.10	104.03	105.25
	**PDE of Neu5Gc**						
Bond	O2-H	O3-H	O4-H	O5-H	O6-H	O7-H	O10-H
Gas	218.48	222.10	217.84	221.93	227.91	217.21	222.67
Benzene	24.55	28.98	25.70	28.58	34.98	24.56	29.06
Acetic acid	9.60	10.94	9.29	10.76	19.65	7.80	10.55
Ethanol	7.47	8.35	7.70	7.96	17.55	5.65	7.59
Formic acid	13.01	13.51	13.82	14.05	19.86	11.73	13.05
Lactic acid	18.35	19.55	17.60	19.22	26.22	16.17	19.26
Water	9.78	10.60	10.17	10.10	22.11	8.04	9.26
	**PA of Neu5Gc**						
Bond	O2-H	O3-H	O4-H	O5-H	O6-H	O7-H	O10-H
Gas	349.69	335.18	337.41	321.84	317.18	322.18	366.36
Benzene	115.46	119.74	101.89	95.90	80.55	90.27	124.17
Acetic acid	63.65	67.18	53.90	54.12	34.16	62.87	66.19
Ethanol	49.60	51.33	39.32	52.78	20.34	50.54	54.36
Formic acid	50.97	52.44	42.04	54.02	24.23	53.13	52.79
Lactic acid	75.79	77.98	63.25	59.65	40.95	71.48	79.43
Water	47.40	48.75	37.94	49.74	23.80	49.78	48.86
	**ETE of Neu5Gc**						
Bond	O2-H	O3-H	O4-H	O5-H	O6-H	O7-H	O10-H
Gas	59.60	87.74	81.24	100.90	111.54	95.84	57.12
Benzene	81.85	91.99	96.56	105.43	127.18	107.04	77.65
Acetic acid	83.46	81.26	92.89	94.15	123.00	82.44	79.87
Ethanol	97.84	97.00	108.36	95.15	137.17	95.08	93.19
Formic acid	96.61	95.64	106.35	94.60	130.20	93.17	94.83
Lactic acid	82.39	81.41	94.19	99.41	125.11	84.53	79.67
Water	96.37	95.84	106.22	94.35	132.30	92.25	94.39

**Table 6:** Antioxidant mechanism parameters of Neu5Ac. (optimized and thermal correction to enthalpy at the M062X/6-311 + G(d,p) level, Single point energy at the M062X/def2TZVPPD level, kcal/mol).

HAT	BDE of Neu5Ac					
Bond	O2-H	O3-H	O4-H	O5-H	O6-H	O7-H
Gas	108.40	108.26	104.24	108.14	113.67	103.38
Benzene	109.13	109.20	105.84	108.85	114.97	104.85
Acetic acid	109.41	109.24	108.42	110.01	118.39	107.14
Ethanol	107.93	108.57	108.09	108.45	118.04	106.18
Formic acid	108.52	109.09	109.32	109.74	114.48	107.42
Lactic acid	107.70	107.86	105.88	107.66	114.56	104.57
Water	105.33	105.92	105.65	105.38	112.17	107.94
	**PDE of Neu5Ac**					
Bond	O2-H	O3-H	O4-H	O5-H	O6-H	O7-H
Gas	224.53	224.39	220.37	224.27	229.80	219.51
Benzene	33.07	33.13	29.78	32.78	38.90	28.79
Acetic acid	12.24	12.06	11.25	12.84	21.22	9.97
Ethanol	9.10	9.74	9.25	9.62	19.21	7.35
Formic acid	14.68	15.25	15.48	15.89	20.63	13.57
Lactic acid	22.60	22.76	20.78	22.56	29.45	19.47
Water	11.71	12.29	12.02	11.76	18.30	14.32
	**PA of Neu5Ac**					
Bond	O2-H	O3-H	O4-H	O5-H	O6-H	O7-H
Gas	354.54	342.11	340.61	327.11	319.68	327.11
benzene	117.94	121.75	103.89	100.18	81.95	94.44
Acetic acid	65.75	68.15	53.78	69.30	35.28	65.96
Ethanol	50.07	51.18	39.54	52.67	20.76	50.83
Formic acid	50.67	51.98	41.52	53.51	23.54	52.55
Lactic acid	77.73	78.55	64.91	63.05	48.85	73.14
water	49.79	48.11	38.00	49.49	23.82	48.58
	**ETE of Neu5Ac**					
Bond	O2-H	O3-H	O4-H	O5-H	O6-H	O7-H
Gas	68.37	80.65	78.13	95.53	108.49	90.78
Benzene	83.71	79.96	94.46	101.18	125.53	102.93
Acetic acid	82.21	79.63	93.19	79.26	121.66	79.73
Ethanol	97.44	96.96	108.12	95.35	137.09	94.93
Formic acid	96.06	95.32	106.01	96.44	129.15	93.08
Lactic acid	81.43	80.77	92.43	96.06	117.16	82.88
Water	93.54	95.80	105.65	93.89	126.11	97.36

**Table 7:** The ionization potential (IP) values of Neu5Gc and Neu5Ac.

IP	Gas	Benzene	Acetic Acid	Ethanol	Formic Acid	Lactic Acid	Water
Neu5Ac	198.37	168.58	135.72	138.41	132.06	136.56	131.63
Neu5Gc	200.81	172.75	137.51	139.97	134.57	139.84	133.99

## Supplementary Materials

The following are available online at http://www.mdpi.com/1420-3049/24/2/313/s1, Table S1: Molecules bond length and angle parameters of Neu5Gc and Neu5Ac, Table S2: Natural bond orbital of Neu5Gc and Neu5Ac.
