# Supplementary material for: Theoretical Studies on the Electronic Structure Parameters and Reactive Activity of Neu5Gc and Neu5Ac under Food Processing Solvent Environment
Source: Molecules. 2019 Jan 16;24(2):313. doi: 10.3390/molecules24020313 (PMC6359032; doi:10.3390/molecules24020313)
Supplement: Supplementary file 1 [file molecules-24-00313-s001.pdf]

Supplementary Materials

Theoretical Studies on the Electronic Structure Parameters and Reactive activity of Neu5Gc and Neu5Ac under food processing solvent environment

Rui Chang[1,2], Bowen Yang[1,2], Qiujin Zhu[1,2*]

1. School of Liquor and Food Engineering, Guizhou University, 2. Key Laboratory of Agricultural and Animal Products Store and Processing of Guizhou Province, Guiyang, 550025, China; cr2011lz@sina.cn (R.C.); patrickyang@zju.edu.cn (B.Y.).
*Corresponding author : ls.qjzhu@gzu.edu.cn , Tel.: + 0851-8292178.

Table 1. Molecules bond length and angle parameters of Neu5Gc and Neu5Ac.

(a) The main bond length parameters of Neu5Gc

Bond	Distance	Bond	Distance	Bond	Distance	Bond	Distance
O1-C13	1.416	O1-C16	1.390	O2-C14	1.406	O3-C17	1.404
O4-C16	1.408	O5-C18	1.423	O6-C19	1.347	O7-C20	1.423
O8-C19	1.191	O9-C21	1.225	O10-C22	1.395	N11-C21	1.344
N11-C12	1.462	C12-C14	1.533	C12-C13	1.535	C13-C17	1.557
C14-C15	1.527	C15-C16	1.526	C16-C19	1.534	C17-C18	1.524
C18-C20	1.515	C21-C22	1.521				

(b) The main bond angle parameters of Neu5Gc

Bond	angle	Bond	angle	Bond	angle	Bond	angle
C13-O1-C16	115.1	O3-C17-C18	109.1	O6-C19-C16	109.4	N11-C12-C13	105.9
O1-C13-C17	106.5	O3-C17-C13	113.4	O6-C19-O8	124.2	N11-C12-C14	112.9
O1-C16-C19	105.6	O4-C16-O1	107.6	O7-C20-C18	105.4	C21-N11-C12	123.2
O1-C16-C15	110.5	O4-C16-C15	112.4	O8-C16-C19	124.2	C13-C12-C14	110.4
O1-C13-C12	111.3	O4-C16-C19	110.2	O9-C21-C22	119.8	C13-C17-C18	110.3
O2-C12-C14	113.5	O5-C18-C20	109.7	O9-C21-N11	123.8	C12-C15-C14	109.5
O2-C14-C15	106.3	O5-C18-C17	106.8	O10-C22-C21	110.8	C16-C15-C14	111.2

(c) The main bond length parameters of Neu5Ac

Bond	Distance	Bond	Distance	Bond	Distance	Bond	Distance
O1-C12	1.420	O1-C16	1.387	O2-C13	1.407	O3-C15	1.406
O4-C16	1.407	O5-C17	1.418	O6-C18	1.347	O7-C19	1.423
O8-C18	1.192	O9-C20	1.221	N10-C20	1.356	N10-C11	1.461

C11-C12	1.534	C11-C13	1.532	C12-C15	1.534	C13-C14	1.526
C14-C16	1.527	C15-C17	1.525	C16-C18	1.534	C17-C19	1.516
C20-C21	1.509						

(d)The main bond angle parameters of Neu5Ac

Bond	angle	Bond	angle	Bond	angle	Bond	angle
C12-O1-C16	115.8	O1-C12-C15	104.3	O1-C16-C14	110.7	O1-C16-C18	105.6
O1-C12-C11	112.3	O2-C13-C11	113.3	O2-C13-C14	107.0	O3-C15-C17	112.4
O3-C15-C12	111.1	O4-C16-O1	107.7	O4-C16-C14	112.2	O4-C16-C18	110.7
O5-C17-C19	110.1	O5-C17-C15	105.6	O6-C18-O8	124.1	O6-C18-C16	109.7
O7-C19-C17	105.6	O9-C20-N10	122.0	O9-C20-C21	121.6	N10-C20-C21	116.3
N10-C11-C13	112.4	N10-C11-C12	106.1	C12-C11-C13	111.0	C11-C13-C14	109.6
C11-C12-C15	112.3	C13-C14-C16	110.8	C12-C15-C17	110.2		

Supplementary material **Table 2.** Natural bond orbital of Neu5Gc and Neu5Ac

(a)Natural bond orbital of Neu5Gc

Donor	Electron density	Receptor	Electron density	E2a	E(j)-E(i)	F(i,j)
σ (O1 - C13)	1.9861	σ* (O3 - C17)	0.0121	0.56	1.32	0.024
σ (O1 - C13)	1.9861	σ* (N11 - C12)	0.0292	1.29	1.34	0.037
σ (O1 - C13)	1.9861	σ* (C16 - C19)	0.0950	1.44	1.33	0.040
σ (O1 - C16)	1.9859	σ* (O4 - H35)	0.0054	1.35	1.47	0.040
σ (O1 - C16)	1.9859	σ* (O6 - C19)	0.0932	1.45	1.38	0.041
σ (O1 - C16)	1.9859	σ* (C13 - C17)	0.0369	1.16	1.38	0.036
σ (O1 - C16)	1.9859	σ* (C15 - H27)	0.0107	1.05	1.45	0.035
σ (O2 - C14)	1.9918	σ* (C12 - C13)	0.0300	1.15	1.34	0.035
σ (O2 - C14)	1.9918	σ* (C15 - C16)	0.0404	1.48	1.33	0.040
σ (O2 - H31)	1.9879	σ* (C12 - C14)	0.0390	0.64	1.23	0.025
σ (O2 - H31)	1.9879	σ* (C14 - C15)	0.0245	2.05	1.24	0.045
σ (O3 - C17)	1.9912	σ* (O1 - C13)	0.0240	0.65	1.29	0.026
σ (O3 - C17)	1.9912	σ* (O5 - C18)	0.0230	1.84	1.29	0.044
σ (O3 - H34)	1.9880	σ* (C13 - C17)	0.0369	1.18	1.21	0.034
σ (O3 - H34)	1.9880	σ* (C17 - C18)	0.0336	0.72	1.25	0.027
σ (O3 - H34)	1.9880	σ* (C17 - H28)	0.0349	0.97	1.24	0.031
σ (O4 - C16)	1.9895	σ* (O8 - C19)	0.0211	1.04	1.67	0.037
σ (O4 - C16)	1.9895	π* (O8 - C19)	0.0211	1.03	1.04	0.030

σ (O4 - C16)	1.9895	σ* (C15 - H26)	0.0129	1.09	1.44	0.035
σ (O4 - H35)	1.9811	σ* (O1 - C16)	0.0504	4.33	1.20	0.065
σ (O5 - C18)	1.9913	σ* (O3 - C17)	0.0121	2.09	1.31	0.047
σ (O5 - C18)	1.9913	σ* (C20 - H33)	0.0221	0.94	1.39	0.032
σ (O5 - H36)	1.9891	σ* (C17 - C18)	0.0336	2.01	1.26	0.045
σ (O6 - C19)	1.9951	σ* (O1 - C16)	0.0504	1.20	1.44	0.037
σ (O6 - C19)	1.9951	σ* (O8 - C19)	0.0211	0.57	1.76	0.028
σ (O6 - H39)	1.9878	σ* (O8 - C19)	0.0211	0.90	1.57	0.034
σ (O6 - H39)	1.9878	σ* (C16 - C19)	0.0950	3.59	1.26	0.061
σ (O7 - C20)	1.9946	σ* (C17 - C18)	0.0336	1.52	1.37	0.041
σ (O7 - H40)	1.9901	σ* (C18 - C20)	0.0276	1.72	1.27	0.042
π (O8 - C19)	1.9963	σ* (C16 - C19)	0.0950	1.51	1.62	0.045
π (O8 - C19)	1.9963	σ* (O4 - C16)	0.0564	1.84	0.83	0.035
π (O8 - C19)	1.9924	π* (O8 - C19)	0.1650	0.53	0.55	0.016
π (O8 - C19)	1.9924	σ* (C15 - C16)	0.0404	0.98	0.91	0.027
σ (O9 - C21)	1.9929	σ* (N11 - C21)	0.0595	1.31	1.71	0.043
σ (O9 - C21)	1.9929	σ* (N11 - H30)	0.0300	1.91	1.63	0.050
σ (O9 - C21)	1.9929	σ* (C21 - C22)	0.0654	0.72	1.59	0.031
π (O9 - C21)	1.9929	σ* (O2 - H31)	0.0267	1.19	1.02	0.031
π (O9 - C21)	1.9887	π* (O9 - C21)	0.2929	1.24	0.54	0.025
π (O9 - C21)	1.9887	σ* (C22 - H37)	0.0232	1.18	0.91	0.029
π (O9 - C21)	1.9887	σ* (C22 - H38)	0.0278	1.15	0.91	0.029
σ (O10 - C22)	1.9950	σ* (N11 - C21)	0.0595	2.13	1.48	0.051
σ (O10 - H41)	1.9880	σ* (N11 - C21)	0.0595	0.58	1.35	0.025
σ (O10 - H41)	1.9880	σ* (C21 - C22)	0.0654	1.65	1.22	0.041
σ (O10 - H41)	1.9880	σ* (C22 - H37)	0.0232	1.22	1.23	0.035
σ (N11 - C12)	1.9812	σ* (O1 - C13)	0.0241	2.55	1.19	0.049
σ (N11 - C12)	1.9812	σ* (N11 - C21)	0.0595	1.65	1.38	0.043
σ (N11 - C12)	1.9812	σ* (C14 - C15)	0.0246	1.17	1.27	0.034
σ (N11 - C12)	1.9812	σ* (C21 - C22)	0.0654	2.43	1.26	0.050
σ (N11 - C21)	1.9891	σ* (O9 - C21)	0.0258	1.19	1.59	0.039
σ (N11 - C21)	1.9891	σ* (O10 - C22)	0.0045	0.54	1.34	0.024
σ (N11 - C21)	1.9891	σ* (N11 - C12)	0.0292	1.67	1.34	0.042
σ (N11 - C21)	1.9891	σ* (N11 - H30)	0.0300	0.92	1.42	0.032
σ (N11 - C21)	1.9891	σ* (C12 - C13)	0.0300	0.65	1.36	0.027
σ (N11 - H30)	1.9806	σ* (O1 - C13)	0.0241	0.8	1.1	0.027
σ (N11 - H30)	1.9806	σ* (O9 - C21)	0.0258	5.86	1.39	0.081

σ (N11 - H30)	1.9806	σ* (N11 - C21)	0.0595	0.63	1.29	0.026
σ (N11 - H30)	1.9806	σ* (C12 - C13)	0.0300	0.5	1.16	0.022
σ (N11 - H30)	1.9806	σ* (C12 - C14)	0.0390	0.51	1.17	0.022
σ (N11 - H30)	1.9806	σ* (C12 - H23)	0.0285	0.61	1.2	0.024
σ (C12 - C13)	1.9731	σ* (O2 - C14)	0.0181	2.76	1.11	0.049
σ (C12 - C13)	1.9731	σ* (O3 - C17)	0.0121	0.56	1.09	0.022
σ (C12 - C13)	1.9731	σ* (N11 - C21)	0.0595	3.05	1.26	0.056
σ (C12 - C13)	1.9731	σ* (C12 - C14)	0.0390	0.99	1.14	0.030
σ (C12 - C13)	1.9731	σ* (C12 - H23)	0.0285	0.63	1.16	0.024
σ (C12 - C13)	1.9731	σ* (C13 - C17)	0.0369	0.83	1.11	0.027
σ (C12 - C13)	1.9731	σ* (C17 - C18)	0.0336	0.54	1.15	0.022
σ (C12 - C14)	1.9749	σ* (N11 - H30)	0.0300	1.3	1.17	0.035
σ (C12 - C14)	1.9749	σ* (C12 - C13)	0.0300	1.29	1.12	0.034
σ (C12 - C14)	1.9749	σ* (C13 - C17)	0.0369	2.1	1.1	0.043
σ (C12 - C14)	1.9749	σ* (C14 - C15)	0.0246	1.02	1.14	0.030
σ (C12 - C14)	1.9749	σ* (C15 - H27)	0.0107	1.7	1.16	0.040
σ (C12 - H23)	1.9735	σ* (O2 - C14)	0.0181	0.79	0.98	0.025
σ (C12 - H23)	1.9735	σ* (N11 - H30)	0.0300	1.83	1.06	0.039
σ (C12 - H23)	1.9735	σ* (C13 - H24)	0.0295	2.97	1.03	0.05
σ (C12 - H23)	1.9735	σ* (C14 - H25)	0.0380	2.54	1.03	0.046
σ (C13 - C17)	1.9763	σ* (O1 - C16)	0.0504	3.17	1.06	0.052
σ (C13 - C17)	1.9763	σ* (O3 - H34)	0.0158	0.52	1.22	0.022
σ (C13 - C17)	1.9763	σ* (C12 - C13)	0.0300	0.92	1.11	0.029
σ (C13 - C17)	1.9763	σ* (C12 - C14)	0.0390	1.73	1.12	0.040
σ (C13 - C17)	1.9763	σ* (C17 - C18)	0.0336	0.7	1.13	0.025
σ (C13 - C17)	1.9763	σ* (C18 - C20)	0.0276	1.88	1.13	0.041
σ (C13 - H24)	1.9799	σ* (O3 - C17)	0.0121	1.28	0.97	0.032
σ (C13 - H24)	1.9799	σ* (N11 - C12)	0.0292	0.62	0.99	0.022
σ (C13 - H24)	1.9799	σ* (C12 - H23)	0.0285	3.3	1.05	0.053
σ (C13 - H24)	1.9799	σ* (C17 - C18)	0.0336	0.53	1.03	0.021
σ (C13 - H24)	1.9799	σ* (C17 - H28)	0.0349	0.62	1.03	0.023
σ (C14 - C15)	1.9725	σ* (O2 - H31)	0.0267	1.68	1.23	0.041
σ (C14 - C15)	1.9725	σ* (N11 - C12)	0.0292	3.28	1.08	0.053
σ (C14 - C15)	1.9725	σ* (C12 - C14)	0.0390	0.85	1.12	0.028
σ (C14 - C15)	1.9725	σ* (C15 - C16)	0.0404	0.9	1.1	0.028
σ (C14 - C15)	1.9725	σ* (C15 - H26)	0.0129	0.59	1.15	0.023
σ (C14 - C15)	1.9725	σ* (C15 - H27)	0.0107	0.63	1.15	0.024

σ (C14 - C15)	1.9725	σ* (C16 - C19)	0.0950	2.35	1.07	0.046
σ (C14 - H25)	1.9754	σ* (N11 - C12)	0.0292	0.85	0.96	0.026
σ (C14 - H25)	1.9754	σ* (C12 - H23)	0.0285	3.26	1.02	0.051
σ (C14 - H25)	1.9754	σ* (C15 - H26)	0.0129	2.91	1.03	0.049
σ (C15 - C16)	1.9740	σ* (O2 - C14)	0.0181	1.93	1.12	0.042
σ (C15 - C16)	1.9740	σ* (O8 - C19)	0.0211	1.48	1.42	0.041
σ (C15 - C16)	1.9740	π* (O8 - C19)	0.1650	2.38	0.78	0.040
σ (C15 - C16)	1.9740	σ* (C14 - C15)	0.0246	0.58	1.16	0.023
σ (C15 - C16)	1.9740	σ* (C15 - H27)	0.0107	0.54	1.19	0.023
σ (C15 - C16)	1.9740	σ* (C16 - C19)	0.0950	0.66	1.11	0.025
σ (C15 - H26)	1.9721	σ* (O2 - C14)	0.0181	0.88	0.97	0.026
σ (C15 - H26)	1.9721	σ* (O4 - C16)	0.0564	5.84	0.91	0.066
σ (C15 - H26)	1.9721	σ* (C14 - H25)	0.0380	3	1.02	0.05
σ (C15 - H27)	1.9742	σ* (O1 - C16)	0.0504	5.08	0.95	0.062
σ (C15 - H27)	1.9742	σ* (O2 - C14)	0.0181	0.54	0.97	0.021
σ (C15 - H27)	1.9742	σ* (C12 - C14)	0.0390	2.89	1.01	0.048
σ (C15 - H27)	1.9742	σ* (C14 - C15)	0.0246	0.61	1.02	0.022
σ (C16 - C19)	1.9712	σ* (O1 - C13)	0.0241	3.73	1.10	0.057
σ (C16 - C19)	1.9712	σ* (O6 - H39)	0.0091	2.58	1.20	0.050
σ (C16 - C19)	1.9712	σ* (O8 - C19)	0.0211	1.55	1.44	0.042
σ (C16 - C19)	1.9712	σ* (C14 - C15)	0.0246	1.83	1.18	0.042
σ (C16 - C19)	1.9712	σ* (C15 - C16)	0.0404	0.84	1.16	0.028
σ (C17 - C18)	1.9764	σ* (O1 - C13)	0.0241	0.51	1.06	0.021
σ (C17 - C18)	1.9764	σ* (O3 - H34)	0.0158	0.75	1.22	0.027
σ (C17 - C18)	1.9764	σ* (O5 - H36)	0.0104	1.92	1.20	0.043
σ (C17 - C18)	1.9764	σ* (O7 - C20)	0.0103	1.9	1.05	0.040
σ (C17 - C18)	1.9764	σ* (C12 - C13)	0.0300	0.9	1.12	0.028
σ (C17 - C18)	1.9764	σ* (C13 - C17)	0.0369	0.66	1.10	0.024
σ (C17 - C18)	1.9764	σ* (C18 - C20)	0.0276	0.73	1.14	0.026
σ (C17 - H28)	1.9790	σ* (O1 - C13)	0.0241	1.16	0.93	0.029
σ (C17 - H28)	1.9790	σ* (O3 - H34)	0.0158	1.18	1.10	0.032
σ (C17 - H28)	1.9790	σ* (C13 - H24)	0.0295	0.8	1.03	0.026
σ (C17 - H28)	1.9790	σ* (C18 - H29)	0.0341	3.07	1.02	0.050
σ (C18 - C20)	1.9816	σ* (O7 - H40)	0.0053	2.13	1.21	0.045
σ (C18 - C20)	1.9816	σ* (C13 - C17)	0.0369	2.16	1.11	0.044
σ (C18 - C20)	1.9816	σ* (C17 - C18)	0.0336	0.97	1.15	0.030
σ (C18 - C20)	1.9816	σ* (C20 - H33)	0.0221	0.5	1.16	0.022

σ (C18 - H29)	1.9765	σ* (O7 - C20)	0.0103	0.57	0.92	0.020
σ (C18 - H29)	1.9765	σ* (C17 - H28)	0.0349	2.96	1.01	0.049
σ (C18 - H29)	1.9765	σ* (C20 - H32)	0.0255	2.69	1.01	0.047
σ (C20 - H32)	1.9871	σ* (C18 - H29)	0.0341	2.91	1.03	0.049
σ (C20 - H33)	1.9838	σ* (O5 - C18)	0.0230	4.85	0.94	0.060
σ (C21 - C22)	1.9800	σ* (O9 - C21)	0.0258	0.72	1.36	0.028
σ (C21 - C22)	1.9800	σ* (O10 - H41)	0.0164	0.59	1.24	0.024
σ (C21 - C22)	1.9800	σ* (N11 - C12)	0.0292	4.84	1.12	0.066
σ (C22 - H37)	1.9748	σ* (O9 - C21)	0.0258	2.16	1.23	0.046
σ (C22 - H37)	1.9748	π* (O9 - C21)	0.2929	5.85	0.65	0.059
σ (C22 - H37)	1.9748	σ* (O10 - H41)	0.0164	1.3	1.11	0.034
σ (C22 - H38)	1.9714	σ* (O9 - C21)	0.0258	2.42	1.23	0.049
σ (C22 - H38)	1.9714	π* (O9 - C21)	0.2929	5.09	0.65	0.055

(b)Natural bond orbital of Neu5Ac

Donor	Electron density	Receptor	Electron density	E2a	E(j)-E(i)	F(i,j)
σ (O1 - C12)	1.9853	σ* (N10 - C11)	0.0284	1.27	1.33	0.037
σ (O1 - C12)	1.9853	σ* (C15 - H27)	0.0279	1.18	1.38	0.036
σ (O1 - C12)	1.9853	σ* (C16 - C18)	0.0946	1.49	1.32	0.040
σ (O1 - C16)	1.9862	σ* (O4 - H34)	0.0057	1.35	1.47	0.040
σ (O1 - C16)	1.9862	σ* (O6 - C18)	0.0936	1.42	1.38	0.040
σ (O1 - C16)	1.9862	σ* (C12 - C15)	0.0370	0.95	1.40	0.033
σ (O1 - C16)	1.9862	σ* (C14 - H25)	0.0109	1.04	1.45	0.035
σ (O2 - C13)	1.9919	σ* (C11 - C12)	0.0321	1.18	1.34	0.036
σ (O2 - C13)	1.9919	σ* (C14 - C16)	0.0411	1.42	1.33	0.039
σ (O2 - H30)	1.9882	σ* (C11 - C13)	0.0381	0.72	1.23	0.027
σ (O2 - H30)	1.9882	σ* (C13 - C14)	0.0254	1.89	1.24	0.043
σ (O3 - C15)	1.9919	σ* (O5 - C17)	0.0214	1.4	1.30	0.038
σ (O3 - C15)	1.9919	σ* (C12 - H23)	0.0287	1.04	1.40	0.034
σ (O3 - H33)	1.9877	σ* (C15 - H27)	0.0279	2.53	1.25	0.050
σ (O4 - C16)	1.9898	σ* (O8 - C18)	0.0217	1.08	1.67	0.038
σ (O4 - C16)	1.9898	π* (O8 - C18)	0.1648	0.9	1.04	0.028
σ (O4 - C16)	1.9898	σ* (C14 - H26)	0.0130	1.09	1.44	0.035
σ (O4 - H34)	1.9812	σ* (O1 - C16)	0.0495	4.25	1.20	0.064
σ (O5 - C17)	1.9904	σ* (O3 - C15)	0.0163	2.49	1.30	0.051
σ (O5 - C17)	1.9904	σ* (C19 - H32)	0.0227	0.97	1.38	0.033

σ (O5 - H35)	1.9886	σ* (C15 - C17)	0.0379	2.11	1.25	0.046
σ (O6 - C18)	1.9952	σ* (O1 - C16)	0.0495	1.12	1.44	0.036
σ (O6 - C18)	1.9952	σ* (O8 - C18)	0.0217	0.55	1.76	0.028
σ (O6 - H39)	1.9877	σ* (O8 - C18)	0.0217	0.93	1.56	0.034
σ (O6 - H39)	1.9877	σ* (C16 - C18)	0.0946	3.57	1.26	0.061
σ (O7 - C19)	1.9946	σ* (C15 - C17)	0.0379	1.48	1.37	0.04
σ (O7 - H40)	1.9901	σ* (C17 - C19)	0.0287	1.73	1.27	0.042
σ (O8 - C18)	1.9963	σ* (C16 - C18)	0.0946	1.48	1.62	0.045
π (O8 - C18)	1.9927	σ* (O4 - C16)	0.0574	1.65	0.84	0.034
π (O8 - C18)	1.9927	π* (O8 - C18)	0.1648	0.54	0.56	0.016
π (O8 - C18)	1.9927	σ* (C14 - C16)	0.0411	0.95	0.91	0.026
σ (O9 - C20)	1.9925	π* (O9 - C20)	0.2625	0.52	1.22	0.024
σ (O9 - C20)	1.9925	σ* (N10 - C20)	0.0681	0.97	1.65	0.036
σ (O9 - C20)	1.9925	σ* (N10 - H29)	0.0171	1.68	1.57	0.046
σ (O9 - C20)	1.9925	σ* (C20 - C21)	0.0484	1.17	1.55	0.038
σ (O9 - C20)	1.9892	σ*(O2 - H30)	0.0272	1.4	1.05	0.034
σ (O9 - C20)	1.9892	π* (O9 - C20)	0.2625	1.63	0.61	0.03
σ (O9 - C20)	1.9892	σ* (C21 - H36)	0.0049	0.84	0.96	0.025
σ (N10 - C11)	1.9821	σ* (O1 - C12)	0.0284	2.66	1.19	0.050
σ (N10 - C11)	1.9821	σ* (N10 - C20)	0.0681	1.47	1.37	0.041
σ (N10 - C11)	1.9821	σ* (C13 - C14)	0.0254	1.21	1.28	0.035
σ (N10 - C11)	1.9821	σ* (C20 - C21)	0.0484	2.52	1.28	0.051
σ (N10 - C20)	1.9902	σ* (O9 - C20)	0.0423	0.77	1.56	0.031
σ (N10 - C20)	1.9902	σ* (N10 - C11)	0.0284	1.61	1.33	0.042
σ (N10 - C20)	1.9902	σ* (N10 - H29)	0.0171	0.59	1.40	0.026
σ (N10 - C20)	1.9902	σ* (C11 - C12)	0.0321	0.69	1.36	0.027
σ (N10 - H29)	1.9843	σ* (O1 - C12)	0.0284	0.83	1.10	0.027
σ (N10 - H29)	1.9843	σ* (O9 - C20)	0.0423	4.37	1.37	0.070
σ (N10 - H29)	1.9843	π* (O9 - C20)	0.2625	0.64	0.86	0.022
σ (N10 - H29)	1.9843	σ* (C11 - H22)	0.0277	0.83	1.21	0.028
σ (C11 - C12)	1.9713	σ* (O2 - C13)	0.0186	2.76	1.10	0.049
σ (C11 - C12)	1.9713	σ* (N10 - C20)	0.0681	3.05	1.24	0.055
σ (C11 - C12)	1.9713	σ* (C11 - C13)	0.0381	0.99	1.14	0.030
σ (C11 - C12)	1.9713	σ* (C11 - H22)	0.0277	0.71	1.17	0.026
σ (C11 - C12)	1.9713	σ* (C12 - C15)	0.0370	1.04	1.13	0.031
σ (C11 - C12)	1.9713	σ* (C15 - C17)	0.0379	1.39	1.15	0.036
σ (C11 - C13)	1.9749	σ* (N10 - H29)	0.0171	0.92	1.16	0.029

σ (C11 - C13)	1.9749	σ* (C11 - C12)	0.0321	1.32	1.12	0.034
σ (C11 - C13)	1.9749	σ* (C12 - C15)	0.0370	1.94	1.12	0.042
σ (C11 - C13)	1.9749	σ* (C13 - C14)	0.0254	1.04	1.14	0.031
σ (C11 - C13)	1.9749	σ* (C14 - H25)	0.0109	1.73	1.16	0.040
σ (C11 - H22)	1.9720	σ* (O2 - C13)	0.0186	0.83	0.97	0.025
σ (C11 - H22)	1.9720	σ* (N10 - H29)	0.0171	2.42	1.04	0.045
σ (C11 - H22)	1.9720	σ* (C11 - C12)	0.0321	0.52	1.00	0.020
σ (C11 - H22)	1.9720	σ* (C12 - H23)	0.0287	3.04	1.04	0.050
σ (C11 - H22)	1.9720	σ* (C13 - H24)	0.0380	2.58	1.03	0.046
σ (C12 - C15)	1.9751	σ* (O1 - C16)	0.0495	3.27	1.08	0.053
σ (C12 - C15)	1.9751	σ* (C11 - C12)	0.0321	1.06	1.12	0.031
σ (C12 - C15)	1.9751	σ* (C11 - C13)	0.0381	1.79	1.13	0.040
σ (C12 - C15)	1.9751	σ* (C12 - H23)	0.0287	0.53	1.16	0.022
σ (C12 - C15)	1.9751	σ* (C15 - C17)	0.0379	0.73	1.14	0.026
σ (C12 - C15)	1.9751	σ* (C15 - H27)	0.0279	0.54	1.14	0.022
σ (C12 - C15)	1.9751	σ* (C17 - C19)	0.0287	1.80	1.15	0.041
σ (C12 - H23)	1.9754	σ* (O3 - C15)	0.0163	3.66	0.97	0.053
σ (C12 - H23)	1.9754	σ* (N10 - C11)	0.0287	0.76	0.99	0.024
σ (C12 - H23)	1.9754	σ* (C11 - H22)	0.0277	3.10	1.05	0.051
σ (C13 - C14)	1.9727	σ* (O2 - H30)	0.0272	1.48	1.23	0.038
σ (C13 - C14)	1.9727	σ* (N10 - C11)	0.0287	3.23	1.08	0.053
σ (C13 - C14)	1.9727	σ* (C11 - C13)	0.0381	0.84	1.12	0.027
σ (C13 - C14)	1.9727	σ* (C14 - C16)	0.0411	0.91	1.10	0.028
σ (C13 - C14)	1.9727	σ* (C14 - H25)	0.0109	0.65	1.15	0.025
σ (C13 - C14)	1.9727	σ* (C14 - H26)	0.0130	0.6	1.15	0.024
σ (C13 - C14)	1.9727	σ* (C16 - C18)	0.0946	2.3	1.07	0.045
σ (C13 - H24)	1.9753	σ* (N10 - C11)	0.0284	0.85	0.96	0.026
σ (C13 - H24)	1.9753	σ* (C11 - H22)	0.0277	3.23	1.02	0.051
σ (C13 - H24)	1.9753	σ* (C14 - H26)	0.0130	2.98	1.03	0.050
σ (C14 - C16)	1.9728	σ* (O2 - C13)	0.0186	2.08	1.12	0.043
σ (C14 - C16)	1.9728	σ* (O8 - C18)	0.0217	1.38	1.41	0.039
σ (C14 - C16)	1.9728	π* (O8 - C18)	0.1648	2.64	0.78	0.042
σ (C14 - C16)	1.9728	σ* (C13 - C14)	0.0254	0.58	1.16	0.023
σ (C14 - C16)	1.9728	σ* (C14 - H25)	0.0109	0.53	1.19	0.022
σ (C14 - C16)	1.9728	σ* (C16 - C18)	0.0946	0.65	1.11	0.024
σ (C14 - H25)	1.9744	σ* (O1 - C16)	0.0495	5.02	0.96	0.062

σ (C14 - H25)	1.9744	σ* (O2 - C13)	0.0186	0.56	0.97	0.021
σ (C14 - H25)	1.9744	σ* (C11 - C13)	0.0381	2.88	1.01	0.048
σ (C14 - H25)	1.9744	σ* (C13 - C14)	0.0254	0.63	1.02	0.023
σ (C14 - H26)	1.9724	σ* (O2 - C13)	0.0186	0.8	0.97	0.025
σ (C14 - H26)	1.9724	σ* (O4 - C16)	0.0574	5.75	0.91	0.065
σ (C14 - H26)	1.9724	π* (C13 - H24)	0.0380	3	1.02	0.050
σ (C15 - C17)	1.9760	σ* (O5 - H35)	0.0093	1.94	1.21	0.043
σ (C15 - C17)	1.9760	σ* (O7 - C19)	0.0109	1.99	1.04	0.041
σ (C15 - C17)	1.9760	σ* (C11 - C12)	0.0321	2.08	1.12	0.043
σ (C15 - C17)	1.9760	σ* (C12 - C15)	0.0370	0.8	1.12	0.027
σ (C15 - C17)	1.9760	σ* (C15 - H27)	0.0279	0.52	1.14	0.022
σ (C15 - C17)	1.9760	σ* (C17 - C19)	0.0287	0.69	1.14	0.025
σ (C15 - H27)	1.9706	σ* (O1 - C12)	0.0284	3.53	0.93	0.051
σ (C15 - H27)	1.9706	σ* (O3 - H33)	0.0131	2.67	1.09	0.048
σ (C15 - H27)	1.9706	σ* (O5 - C17)	0.0214	0.74	0.94	0.024
σ (C15 - H27)	1.9706	σ* (C17 - H28)	0.0350	3.01	1.03	0.050
σ (C16 - C18)	1.9712	σ* (O1 - C12)	0.0284	3.85	1.10	0.058
σ (C16 - C18)	1.9712	σ* (O6 - H39)	0.0093	2.65	1.19	0.050
σ (C16 - C18)	1.9712	σ* (O8 - C18)	0.0217	1.54	1.44	0.042
σ (C16 - C18)	1.9712	σ* (C13 - C14)	0.0254	1.86	1.18	0.042
σ (C16 - C18)	1.9712	σ* (C14 - C16)	0.0411	0.79	1.16	0.027
σ (C17 - C19)	1.9820	σ* (O7 - H40)	0.0053	2.10	1.21	0.045
σ (C17 - C19)	1.9820	σ* (C12 - C15)	0.0370	2.14	1.12	0.044
σ (C17 - C19)	1.9820	σ* (C15 - C17)	0.0379	0.98	1.14	0.030
σ (C17 - H28)	1.9771	σ* (O7 - C19)	0.0109	0.56	0.92	0.020
σ (C17 - H28)	1.9771	σ* (C15 - H27)	0.0279	2.89	1.01	0.048
σ (C17 - H28)	1.9771	σ* (C19 - H31)	0.0255	2.72	1.01	0.047
σ (C19 - H31)	1.9874	σ* (C17 - H28)	0.0350	2.86	1.04	0.049
σ (C19 - H32)	1.9844	σ* (O5 - C17)	0.0215	4.66	0.95	0.059
σ (C20 - C21)	1.9833	σ* (O9 - C20)	0.0423	0.98	1.35	0.033
σ (C20 - C21)	1.9833	σ* (N10 - C11)	0.0284	4.74	1.13	0.065
σ (C21 - H36)	1.9735	π* (O9 - C20)	0.2625	5.43	0.69	0.058
σ (C21 - H36)	1.9735	σ* (N10 - C20)	0.0681	1.56	1.12	0.038
σ (C21 - H37)	1.9787	σ* (O9 - C20)	0.0423	0.57	1.21	0.024
σ (C21 - H37)	1.9787	σ* (O9 - C20)	0.2625	3.42	0.69	0.046
σ (C21 - H37)	1.9787	σ* (N10 - C20)	0.0681	2.61	1.12	0.049
σ (C21 - H38)	1.9885	σ* (O9 - C20)	0.0423	4.74	1.22	0.068

σ (C21 - H38)	1.9885	π* (O9 - C20)	0.2625	1.36	0.70	0.030
σ (C21 - H38)	1.9885	σ* (N10 - C20)	0.0681	0.59	1.13	0.024
